# Exploring the shifting landscape of delayed motherhood in India: a comprehensive analysis using joinpoint and age-period-cohort analysis

**DOI:** 10.1186/s12905-025-04104-4

**Published:** 2025-11-19

**Authors:** Mayank Singh, Chander Shekhar, Jagriti Gupta, Soumen Barik

**Affiliations:** 1 Department of Epidemiology and Biostatistics, KAHER, Belgaum Karnataka, 590010 India; 2 Department of Fertility & Social Demography, International Institute for Population Sciences (IIPS), Mumbai, 400088 India

**Keywords:** Delayed Motherhood, Age-Period-Cohort Analysis, Joinpoint Regression, National Family Health Survey, India

## Abstract

**Introduction:**

The increasing trend of delayed motherhood in both developed and developing countries has led to demographic shifts in fertility patterns. In India, the transition towards later childbearing has been largely unexplored. This study aims to investigate the trends in delayed motherhood prevalence, along with socio-economic and demographic factors contributing to this shift.

**Data and methods:**

This study utilizes data from five rounds of the National Family Health Survey (NFHS), with a focus on ever-married women aged 40–49 years. Delayed motherhood is defined as childbirth occurring at or beyond 35 years of age. The analysis employed joinpoint regression and age-period-cohort (APC) modelling to assess the trends, examining the effects of socio-economic, educational, and demographic factors on delayed motherhood.

**Results:**

The findings indicate a significant increase in delayed motherhood with age, particularly for women aged 48, where the prevalence was highest at 15.14%. Period effect analysis shows a steady decline in delayed motherhood prevalence since 1992, but variations exist across socio-economic groups. Women with secondary education and from wealthier households exhibit higher risks of delayed motherhood. APC analysis reveals a general decline in the risk of delayed motherhood in newer cohorts, though this trend is less evident among women from lower socio-economic backgrounds and certain religious groups.

**Conclusion:**

The demographic shift toward delayed motherhood in India poses both challenges and opportunities for public health. Socio-economic disparities in access to healthcare and education are critical factors influencing this trend. Policy interventions aimed at addressing these disparities could mitigate the risks associated with delayed maternal age and improve maternal and child health outcomes.

**Supplementary Information:**

The online version contains supplementary material available at 10.1186/s12905-025-04104-4.

## Introduction

In recent years, a decline in the age at last birth and an increase of delayed first childbearing among women have been observed in both developed and developing countries. These trends together shorten the reproductive lifespan and cause a decline in fertility rates, indicating the start of a demographic shift from high to low fertility within a nation [1, 2]. Research indicates that in the 1970 s, Indian women started having children at young ages. The average age at last birth ranged from 39 to 42 years old, which increased fertility rates [3]. Delayed motherhood refers to the continuation of childbearing beyond age 35, irrespective of whether the woman has already had previous children [4] [5]. Over the last few decades (1992–1993 to 2019–2021), India has witnessed a substantial decline in the mean age at last birth, from 30.6 years in NFHS-1 to 27.4 years in NFHS-5 [6]. Drawing on NFHS-5 and LASI Wave 1 data, the estimated mean age at natural menopause in India is 46 years, ranging from 44 years in Bihar to 47.6 years in Kerala [7]. Different personal, societal, and economic aspects, these include the financial burden of raising a child, the accessibility of high-quality, affordable childcare, the financial impact of having to take time off work to raise children, cultural and ethnic norms, personal views about the ideal environment in which to raise children, and perceptions of one's individual willingness for parenthood family planning techniques, behavioural modifications, and sociocultural advancements, that lead to several nations have seen a fall in the number of young women giving birth in recent decades [8–10].

Advanced (delayed) maternal age is associated with several risks, including increased rates of infertility (Te [11]), chromosomal abnormalities like Down syndrome [12, 13], caesarean sections [14, 15] preterm delivery [16, 17], low birthweight [16, 17], stillbirth [18, 19], and perinatal mortality and morbidity. In addition, women over 40 have an increased risk of perinatal death, placenta previa, gestational diabetes, and placental abruption. Nonetheless, there are a few documented benefits to being an older mother. According to [20] and [21], children of older moms may be smarter and more intelligent, receive less physical abuse and be more independent [22]. Furthermore, a decline in infant mortality and sudden infant death syndrome has been seen [23].

Although the factors contributing to the variability in delayed pregnancy over the decade are not well-studied, researchers suggest that the decision to delay childbearing is influenced by social factors and individual choices, often related to constraints on family size [24]. The average age at which women become pregnant has increased in many industrialized nations over the last thirty years, partly as a result of changing roles and expectations for women [25]. An increasing number of women in India are also choosing to delay marriage [26]. Although urban populations in India are increasingly aware of age-related fertility decline due to widespread media and education, delayed motherhood persists as a result of socioeconomic and cultural factors rather than lack of knowledge [27] [28]. Women’s decisions to delay childbearing are often shaped by education, career aspirations, and financial stability rather than misinformation [29] [10]. In contrast, rural populations may still face gaps in reproductive health awareness [30]. Research has repeatedly shown that there is an increased risk of adverse consequences of delayed motherhood for both mother and child. Therefore, the demographic change towards later childbearing is of growing importance in clinical and emerging public health concern [28, 31]. In the Indian context, delayed childbearing is still a largely unexplored topic, with little research available [6] despite an extensive amount of literature on the adverse effects of delayed motherhood on mothers and children. This study aims to fill this gap by examining the prevalence and trends of delayed motherhood in India from 1981 to 2010, using nationally representative data from first five rounds of the National Family Health Survey (NFHS). To disentangle the complex temporal dynamics, we employ joinpoint regression and Age-Period-Cohort (APC) analysis. APC modelling is an established epidemiological method used to assess the independent influences of age, period and cohort on the likelihood of delayed motherhood. This approach allows us to identify critical turning points in trends and to understand how socio-economic, educational, and demographic factors shape the timing of late childbearing in Indian women [32, 33].

## Data and methods

### Data source

The study utilized data from first five rounds of the National Family Health Survey (NFHS), a cross-sectional and nationally representative data source. Each round of NFHS is associated with different time periods in India. For example, NFHS-1 was undertaken during 1992–93 at the national level, covering 88,562 households and 89,777 ever-married women aged 13–49. NFHS-2, conducted in 1998–99, covered more than 90,000 eligible women aged 15–49 from 26 states. NFHS-3 occurred during 2005–06 with a sample of 109,041 households and 124,385 women aged 15–49. NFHS-4, conducted in 2015–16, covered 699,686 women in the age group 15–49 from 30 states with a 97% response rate. The most recent data, NFHS-5, was conducted in two phases from 2019 to 2021, covering 707 districts, 28 states, and 8 union territories, with a large sample size of 636,699 households, 724,115 women, 101,839 men, and 232,920 recent births.

**Fig. 1:**
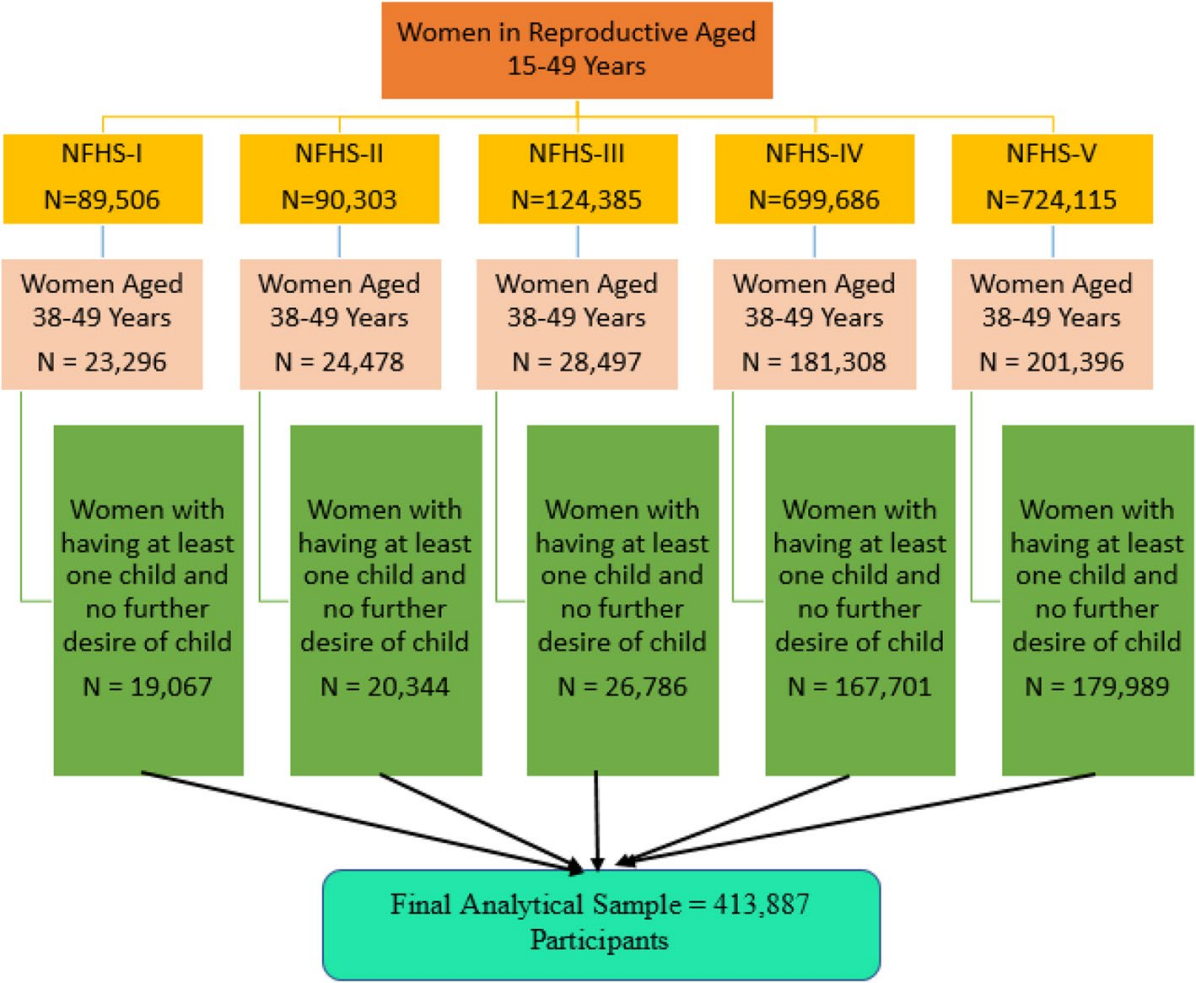
Sample selection process of study participants for the analysis of delayed motherhood in India

For the purpose of examining delayed motherhood, the analysis focused on women aged 38–49 years. This yielded 23,296 women in NFHS-I, 24,478 in NFHS-II, 28,497 in NFHS-III, 181,308 in NFHS-IV, and 201,396 in NFHS-V. From this subset, only those women who had at least one child and expressed no further desire for children were included in the final analytic sample. This resulted in 19,067 participants from NFHS-I, 20,344 from NFHS-II, 26,786 from NFHS-III, 167,701 from NFHS-IV, and 179,989 from NFHS-V. Combining these, the final analytical sample consisted of 413,887 women who met the eligibility criteria and were included in the analysis (Fig. 1).

### Variable description

#### Outcome variable

During the survey, all women who had given birth were asked detailed questions about each childbirth, including the month and year of birth. Additionally, information on the woman’s own month and year of birth was available. Using these variables, we calculated the woman’s age at her most recent (last) birth. Most recent birth was considered as the last birth of the women. In this study, if a woman had her last birth at the age of 35 years or older, she was categorized as experiencing delayed motherhood; otherwise, she was classified as not experiencing delayed motherhood (last birth before 35 years of age).

#### Explanatory variables

The study's socioeconomic and demographic factors for delayed motherhood were classified as follows. According to the survey, the places of residence were rural and urban. Hindu, Muslim, Christian, and Others were recorded as religions. Caste was recorded as Scheduled Caste (SC), Scheduled Tribe (ST), Other Backward Class (OBC), and others. The Wealth Index was divided into four categories: poorest, poorer, middle, richer, and richest. Respondents' educational status was recorded as No education, Primary, Secondary, and Higher. Mass media exposure was assessed using the following questions, i) Do you read a newspaper or magazines almost every day, at least once a week, less than once a week, or not at all? ii) Do you listen the radio almost every day, at least once a week, less than once a week, or not at all? iii) Do you watch television almost every day, at least once a week, less than once a week, or not at all? iv) Do you usually go to a cinema hall or theatre to see a movie at least once a month?. Based on the response the binary classification, if a respondent reported exposure to any of the media sources, she was categorized as having any media exposure; otherwise, she was classified as having no media exposure. India's regions were classified as East, West, North, South, Central, and Northeast. Prior marriage relationship with husband was coded as yes or no. Age at first marriage is reported as marriage under 18, between 18 and 20, and above 20, and previous parity is coded as zero, 1–2, 3–4, or greater than 4.

### Statistical analysis

We have used the bivariate, joinpoint, and age period cohort analysis to fulfill the study’s objective.

### Joinpoint regression analysis

To capture the overall changes in the prevalence of delayed motherhood, we employed joinpoint regression analysis using the joinpoint regression program version 4.5.0.1 (Statistical Research and Applications Branch, National Cancer Institute) as described by [34]. The joinpoint regression technique differs from traditional piecewise or segmented regression models as it automatically identifies significant joinpoint(s) and their location(s) within the model rather than specifying them randomly as in piecewise regression. For each statistically significant segment of the time trend, the model calculates the average percentage change (APC), reflecting the rate of change between two joinpoints. Additionally, the model provides the value of the average annual percentage change (AAPC), representing the overall rate of change in the prevalence of delayed motherhood. This approach allows us to analyze trends and assess the significance of changes over time in a more robust and data-driven manner. To estimate the APC, the following model was used

$$\text{log}(\mathbf{Y}\mathbf{x})=\mathbf{b}0 +\mathbf{b}1\mathbf{x},$$

where log (Y_x_) is the natural logarithm of the rate in year x.

Then the APC from year x to x + 1 is defined as

$$APC=\frac{{e}^{{b}_{0}+{b}_{1}(x+1)}-{e}^{{b}_{0}+{b}_{1}x}}{{e}^{{b}_{0}+{b}_{1}x}}*100=\left({e}^{{b}_{1}}-1\right)*10$$

When there are no joinpoints (APC = AAPC), the trend remains constant. However, if there are joinpoints, the entire time period is divided into segments based on points where the trend changes. The Average Annual Percentage Change (AAPC) is calculated as a geometrically weighted average of the estimated Average Percentage Change (APC) in each segment, with segment lengths serving as weights [35].

The Z-test determines whether an AAPC or APC is significantly different from zero. The terms 'increase' and 'decrease' are used only when the slope (AAPC or APC) of the trend is statistically significant, indicating a significant upward or downward change over time. The term 'stable' refers to a non-significant slope of the trend where there is no significant change.

To calculate the overall trend for the entire study period, we employ the best model with a maximum of 5 join points, creating 6 segments in total. This approach enables us to examine changes in the prevalence of delayed motherhood over time and identify significant trends.

### Age period cohort analysis

Prevalence of delayed motherhood reflects not only the situation experienced by the population in a given year but also the consolidation of various societal and legal changes that happened since birth.

The Age Period Cohort (APC) Analysis is a widely used statistical technique to describe the complex situation of the social, environmental, and historical factors that simultaneously affect individuals and groups of individuals at the same time [36]. In the current paper, APC analysis is used to estimate the net age, period, and cohort effects on delayed motherhood. It is well known that APC suffers from an identification problem because of the linear relational-ship between age, period and cohort i.e. cohort = period – age. Therefore, APC Maximum Entropy (IE) method was used to decompose the temporal trends and provides unbiased, valid and relatively efficient estimation result.

The model was can be represented as:

$$Y=\log\left(M\right)=\mu+\alpha age_i+\beta period_j+\gamma cohort_k+\varepsilon\\$$

where, M stands for the prevalence of delayed motherhood of the corresponding age group, **µ** stands for the intercept item, **α**, **β**, and **γ** stand for the corresponding coefficients of the age, period and cohort effect, and ɛ denotes the residual of the APC model. It has age_1_** = **period_1_—cohort_1_ [37].

Stata 16.0 software (Stata Corp, College Station, TX, USA) was used to run the APC ME (Age Period Cohort Maximum Entropy) model. Deviance, Akaike's information criterion (AIC), and the Bayesian information criterion were used to assess the degree of model fitting (BIC).

## Results

**Fig. 2:**
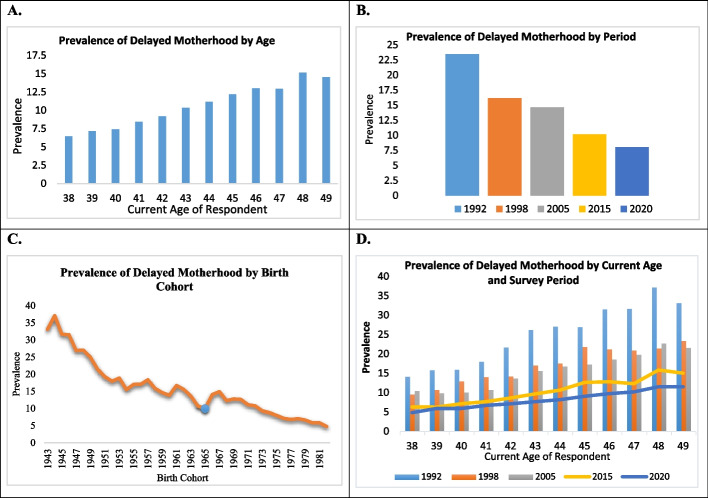
Distribution of delayed motherhood by age, period and birth cohort (Panel A: Prevalence by age; Panel B: Prevalence by period; Panel C: Prevalence by birth cohort; Panel D: Prevalence by combined age and period)

Figure 2 displays the distribution of delayed motherhood by age, period, and birth cohort. Figure A shows the prevalence of delayed motherhood by age. From this figure, it can be observed that women at age 38 have a prevalence of 6.45% for delayed motherhood, while women at age 39 have a prevalence of 7.15%. For women aged 48, the prevalence is 15.14%, and for women aged 49, it is 14.54%. Therefore, it is evident that the prevalence of delayed motherhood increases with age. Figure B presents the prevalence of delayed motherhood by period. This figure indicates that women belonging to the period 1992 have a prevalence of 23.49% for delayed motherhood, whereas those belonging to 1998 have a prevalence of 16.17%. For the periods 2005, 2015, and 2020, the prevalence of delayed motherhood among women aged 40–49 years was 14.64, 10.17, and 8.06, respectively. Figure C illustrates the prevalence of delayed motherhood by birth cohort. It shows that women from the birth cohort of 1943 had a prevalence of 33.01%, which increased to 37.01% for those born in 1945. However, there was a decline in prevalence from the 1944 cohort to 1945 and then from 1946 to 1947. Similarly, the prevalence for the birth cohort of 1965 was 9.99%, but it increased to 14.06% in 1966 and 14.93% in 1967 before gradually declining in subsequent years.

Figure D illustrates the prevalence of delayed motherhood across different age groups and survey periods. Generally, the prevalence of delayed motherhood has decreased over the years for most age groups, except for women aged 38 and 48. For women aged 38, the prevalence initially declined from 14.1% in 1992 to 9.5% in 1998. However, it increased to 10.4% in 2005 before declining again to 4.8% in 2020. Similarly, for women aged 48, the prevalence was 37% in 1992, 21.3% in 1998, 22.6% in 2005, 15.8% in 2015, and then declined to 11.5% in 2020. For the age group 42, the prevalence of delayed motherhood was 21.6% in 1992, 14.2% in 1998, 13.6% in 2005, 8.6% in 2015, and 7.1% in 2020. As for the age group 43, the prevalence was 26.1% in 1992, 17.0% in 1998, 15.6% in 2005, 9.7% in 2015, and 7.7% in 2020. The most significant decline in the prevalence of delayed motherhood occurred between the survey period 1992 and 1998, with the highest decline observed for women aged 48 (42%), followed by ages 43 and 44 with declines of 34% and 35%, respectively. Overall, the age group with the highest decline in prevalence from 1992 to 2020 was the age 43 category, experiencing a 70% decrease, while the lowest decline was seen in the age 39 category, with a 62% decrease.

Table 1 presents the prevalence trend of delayed motherhood in India from 1981 to 2010, analyzed using joinpoint regression. The overall average annual percent change (AAPC) in the prevalence of delayed motherhood was 6.85 (95% CI: 4.41–8.8). This increase in the overall APC was primarily attributed to a higher proportion of older age respondents after the year 2000. However, before 2000, there was a significant decline in delayed motherhood across all background characteristics. The steepest decline in APC for both urban and rural areas was observed at −14.69 (years: 1985 to 1999, 95% CI: −16.87 to −12.45) and −15.66 (years: 1986 to 1995, 95% CI: −17.39 to −13.33), respectively. Regarding educational background, women with no education experienced the highest declining APC of −15.0 (95% CI: −18.08 to −11.81) during the years 1986 to 1994. Similarly, women belonging to the richest category of the wealth index had a declining APC of −14.96 (95% CI: −17.18 to −12.67) between 1985 and 1999. The table also includes the highest declining segments for all other covariates during the entire period from 1980 to 2010. Supplementary Fig. 1 displays the overall prevalence trend of delayed motherhood, showing an initial increase followed by a decline until 2001, and then a gradual increase thereafter. This pattern is observed across all categories except for women with previous parity of 0 and women with previous parity greater than 5. For women with previous parity of 0, the increase in prevalence was observed only after 2001. However, for women with previous parity greater than 5, the prevalence initially increased and peaked at age 50 in 1992, followed by a steep decline from 1992 to 2001, and then a gradual increase afterward.

**Table 1:** Trends in prevalence of delayed motherhood in india from 1981 to 2010 using the joinpoint regression analysis

Characteristics	AAPC* (95% CI)	*P*-Value	Highest Declining Segment	APC	*P*-Value	Characteristics	AAPC* (95% CI)	*P*-Value	Highest Declining Segment	APC	*P*-Value
**Overall**	6.58^a^ [4.41 8.8]	< 0.001	1986–1995	−16.25^a^ [−18.74 −13.68]	< 0.001	**Hindu**	6.90^a^ [4.78 9.06]	< 0.001	1985–1999	−14.37^a^ [−16.03 −12.67]	< 0.001
**Urban**	7.52^a^ [4.72 10.39]	< 0.001	1985–1999	−14.69^a^ [−16.87 −12.45]	< 0.001	**Muslim**	4.62^a^ [2.04 7.27]	< 0.001	1989–2000	−13.54^a^ [−17.02 −9.9]	< 0.001
**Rural**	6.28^a^ [4.32 8.27]	< 0.001	1986–1995	−15.66^a^ [−17.93 −13.33]	< 0.001	**Christian**	5.37^a^ [1.52 9.37]	0.006	1981–1999	−9.98^a^ [−13.41 −6.42]	< 0.001
**No Education**	5.76^a^ [3.71 7.84]	< 0.001	1986–1994	−15.00* [−18.08 −11.81]	< 0.001	**Others**	3.41^a^ [0.25 6.66]	0.034	1981–1998	−15.06^a^ [−18.04 −11.98]	< 0.001
**Primary**	9.53^a^ [5.61 13.59]	< 0.001	1984–2000	−12.54^a^ [−14.78 −10.25]	< 0.001	**SC**	6.39^a^ [4.07 8.76]	< 0.001	1987–1999	−13.34^a^ [−15.95 −10.66]	< 0.001
**Secondary**	10.33^a^ [6.79 13.99]	< 0.001	1984–1999	−14.92^a^ [−17.1 −12.69]	< 0.001	**ST**	5.68^a^ [0.36 11.28]	0.036	1987–1990	−26.23 [−54.01 18.31]	0.194
**Higher**	13.93^a^ [8.4 19.74]	< 0.001	1986–2000	−14.68^a^ [−18.98 −10.15]	< 0.001	**Others**	6.58^a^ [4.6 8.6]	< 0.001	1986–1995	−17.07^a^ [−19.32 −14.75]	< 0.001
**Poorest**	5.64^a^ [3.61 7.71]	< 0.001	1985–2000	−10.91^a^ [−12.41 −9.38]	< 0.001	**No Media**	4.98^a^ [3.45 6.53]	< 0.001	1987–2000	−13.89^a^ [−15.41 −12.33]	< 0.001
**Poorer**	4.93^a^ [2.74 7.18]	< 0.001	1986–1999	−14.65^a^ [−16.75 −12.51]	< 0.001	**Any Media**	8.03^a^ [5.13 11.01]	< 0.001	1984–1999	−12.07^a^ [−13.95 −10.16]	< 0.001
**Middle**	7.34^a^ [4.29 10.48]	< 0.001	1985–1996	−16.27^a^ [−18.45 −14.03]	< 0.001	**East**	6.56^a^ [4.53 8.62]	< 0.001	1986–1999	−13.87* [−15.78 −11.91]	< 0.001
**Richer**	6.26^a^ [2.29 10.38]	0.002	1984–2000	−14.23^a^ [−16.53 −11.87]	< 0.001	**West**	7.38^a^ [3.95 10.91]	< 0.001	1986–1999	−18.43^a^ [−21.47 −15.27]	< 0.001
**Richest**	7.65^a^ [4.78 10.6]	< 0.001	1985–1999	−14.96^a^ [−17.18 −12.67]	< 0.001	**North**	7.08^a^ [5.04 9.17]	< 0.001	1985–2000	−15.59^a^ [−17 −14.15]	< 0.001
**Age at Marriage < 18 years**	*6.01*^a^* [4.19 7.86]*	< 0.001	1986–1994	−15.01^a^ [−17.74 −12.18]	< 0.001	**South**	6.70^a^ [2.91 10.64]	< 0.001	1985–2000	−14.61^a^ [−17.28 −11.86]	< 0.001
**Age at Marriage 18–21 years**	*7.27*^a^* [3.41 11.28]*	< 0.001	1985–1999	−16.33^a^ [−19.29 −13.27]	< 0.001	**Central**	4.44^a^ [2.73 6.17]	< 0.001	1986–2000	−13.39^a^ [−14.86 −11.89]	< 0.001
**Age at Marriage >= 21 years**	*7.05*^a^* [2.08 12.26]*	0.005	1987–2000	−18.09^a^ [−20.16 −15.96]	< 0.001	**North East**	7.46^a^ [3.52 11.55]	< 0.001	1987–2000	−11.52^a^ [−15.45 −7.41]	< 0.001
**Previous Parity Zero**	*13.85*^a^* [3.46 25.3]*	0.008	1981–1996	−10.82 [−22.1 2.08]	0.093	**Previous Parity 3–4**	*7.77*^a^* [3.27 12.47]*	*0.001*	1987–2000	−14.68^a^ [−16.62 −12.69]	< 0.001
**Previous Parity 1–2**	11.76^a^ [4.77 19.2]	0.001	1986–1990	−33.75^a^ [−54.88 −2.72]	0.037	**Previous Parity >= 5**	*3.81*^a^* [2.55 5.07]*	< *0.001*	1992–2001	−10.67^a^ [−12.34 −8.96]	< 0.001

^a^Indicates that the Average Annual Percent Change (AAPC) is significantly different from zero at the alpha = 0.05 level

### Age effect

**Fig. 3:**
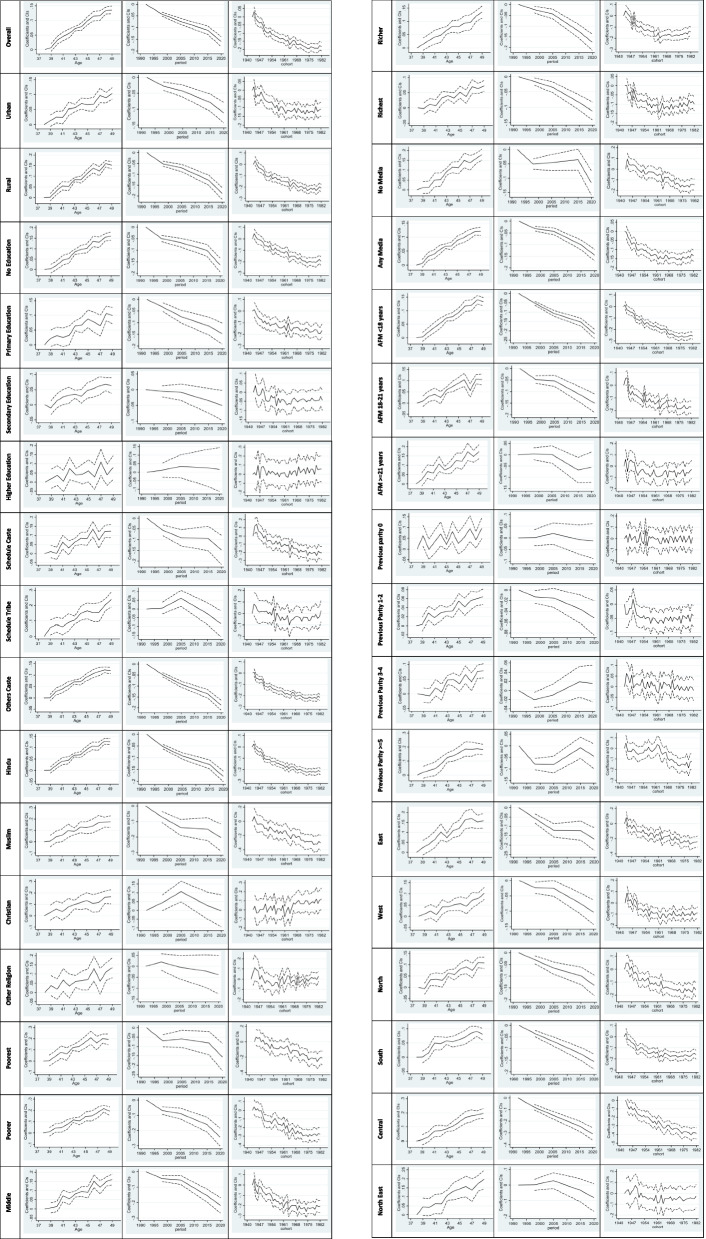
Age, period and cohort effect of delayed motherhood in India

Figure 3 illustrates the age, period, and cohort effects of advance motherhood in India, using women aged 38 as the reference age. The overall age effect analysis reveals that as age increases, the risk of advance motherhood also increases, except for a slight decline observed at the age of 47, followed by an increase towards ages 48 and 49. In general, the overall age effect demonstrates a rising risk of advance motherhood as women age. A similar trend in the age effect of advance motherhood can be observed across all socio-economic and demographic characteristics. For example, women with primary education have the highest risk of advance motherhood at ages 45 and 48, while for women with higher education, the highest risk is found at ages 42 and 47. This pattern can also be seen among the richest group, women with a minimum age at first marriage (AFM) of 21 years, those with a previous parity of 0, and women from the eastern region. In summary, the age effect analysis shows a consistent increase in the risk of advance motherhood as women grow older, with only a slight dip in risk observed at age 47 before rising again in subsequent ages. This pattern is consistent across various socio-economic and demographic characteristics.

### Period effect

From the period effect analysis of delayed motherhood, it becomes evident that over time, the risk of delayed motherhood has significantly declined compared to the period 1992–93. This significant declining trend, with reference to the period 1992–93, is observed across almost all characteristics, except for some specific groups. These exceptions include individuals with secondary education, higher education, SC (Scheduled Caste), ST (Scheduled Tribe), Christian, Other religion, age at first marriage >= 21 years, previous parity 0, and previous parity greater than three. For these characteristics, the declining risk of advance motherhood is not found to be significant.

### Cohort effect

The birth cohort effect analysis reveals a high level of variability and uncertainty in the prevalence of delayed motherhood. However, a clear trend can be observed for most demographic characteristics, indicating a declining risk of delayed motherhood in newer birth cohorts. In other words, women from more recent birth cohorts have a lower risk of giving birth after the age of 35 years compared to older birth cohorts. However, some characteristics do not show a significant declining trend in the risk of delayed motherhood by birth cohort. These characteristics include individuals with secondary education, higher education, ST (Scheduled Tribe), Christian, Other religions, age at first marriage >= 21 years, previous parity 0, previous parity greater than three, and those from the North East region. For these characteristics, the risk of delayed motherhood does not exhibit a clear declining pattern across newer birth cohorts. In summary, while most demographic characteristics demonstrate a declining risk of delayed motherhood in more recent birth cohorts, certain specific characteristics do not show a significant trend in the risk of delayed motherhood over time. The birth cohort effect analysis highlights the complexities and variations in the risk of delayed motherhood across different subgroups in the population.

## Discussion

The study shows notable decrease in delayed motherhood over the previous three decades using data from all five rounds of the National Family Health Survey (NFHS) and joinpoint and Age-Period-Cohort analyses. There were several fluctuations in the prevalence between age groups and birth cohorts, with a peak observed in 1992 and a decline in 2020. An increasing public health risk is highlighted by the trend of delayed motherhood in India as the country moves closer to becoming developed. Managing the complex relationships between socioeconomic factors impacting fertility practices in India requires an understanding of these patterns.

The prevalence of delayed motherhood increases with age, with significant increases observed in women 48 and 49 years of age compared to 38 and 39 years of age. Which indicates that older women had higher prevalence of becoming mother at or after 35 years of age. This is consistent with findings from the study done by Srivastava et al., who observed similar trends in older maternal age groups [38]. According to period analysis, the prevalence peaked in 1992 at 23.49% and declined to 8.06% in 2020. This pattern is consistent with the research of [39], who also saw a high in the early 1990 s and a fall in subsequent years as a result of things like growing awareness of the decline in fertility. According to the cohort analysis, the prevalence increased for birth cohorts born between 1943 and 1947 and then decreased for generations born after 1965. Shows that the prevalence of delayed motherhood has declined over time for most age categories, with the largest drop occurring between 1992 and 1998, especially for women aged 43, who had a 70% fall. In contrast, the age 39 group saw the lowest rate of decline 62%. This overall decrease is consistent with the findings of [6], who noted that within the last three decades, there has been a decline of 15.8% in median age at last birth among women aged 40–49 years caused by enhanced education and mass media reduced reproductive period as women are completing their childbearing at an early age [40].

A shifting trend emerges from the joinpoint regression analysis of delayed motherhood in India between 1981 and 2010. The overall average annual percent change (AAPC) in the prevalence of delayed motherhood was 6.85%. This finding is consistent with other studies, where authors reported the increased fertility control awareness, reduced infant mortality, higher education levels, greater access to contraception, and shortened reproductive lifespans as a result of women ending their childbearing at a younger age [6, 41]. With a notable increase after 2000, attributed to a higher proportion of older women giving birth. This increase is consistent with research by Safdari-Dehcheshmeh et al. [42], which showed an increase in delayed motherhood because of the extension of women's education, labour market participation, medical advancements, and changing cultural norms. Prior to 2000, a significant decline was observed across all background characteristics. In the late 1980 s and early 1990 s, the largest drops in delayed motherhood were observed in rural areas (15.66%) and urban areas (−14.69%). This is supported by (M. Singh et al.) [6] that similar reductions linked to improvements such as education and access to contraception contributed to the observed decline in the delay of motherhood. The wealthiest women and those without formal education saw the sharpest decreases. After declining until 2001, the prevalence of delayed motherhood progressively rose again due to economic stability and career aspirations [27] similarly attributed the increase to socio-cultural shifts [43] the advancement of modern reproductive techniques [44]. With the exception of women who had never given birth or who had given birth more than five times, this tendency was largely constant throughout the majority of the groups. as reported by [45], who identified variations in reproductive practices for these specific groupings, perhaps as a result of unique biological and socioeconomic circumstances [46]. These women showed different patterns of changes in prevalence.

Age effect analysis shows a consistent decrease in the risk of delayed motherhood with recent age, except for a slight decline at age 47. Where the risk briefly declines before rising again at ages 48 and 49. This trend is observed across all socio-economic and demographic characteristics. For instance, women with primary education face the highest risk at ages 45 and 48, while those with higher education have increased risk at ages 42 and 47 a finding consistent with [47]. Similarly, this pattern is evident among the wealthiest women, those who married at 21 years or older, women with no previous children, and those from the eastern region, The wealthiest women have a higher risk of delayed motherhood which aligns with findings from [28].

Period effect analysis indicates a significant overall decline in delayed motherhood risk since 1992–93 [38], with exceptions for specific educational and demographic groups. This decline is evident across nearly all demographic and socio-economic characteristics. However, there are notable exceptions for individuals with secondary or higher education, Scheduled Caste (SC) and Scheduled Tribe (ST) groups; it could be explained by socioeconomic challenges, social norms, a lack of access to healthcare, and disparity of educational attainment lead to no significant decline trend [48]. Christians and those of other religions, women who married at age 21 or older, and women with no previous children or more than three previous children do not show a significant decline in risk because of early parenthood became less desirable due to a number of factors, such as women's empowerment, increased education, and altered relationships within families [29].

The cohort effect analysis reveals a general decline in the risk of delayed motherhood in newer birth cohorts, though some subgroups show no significant trend. Women from more recent birth cohorts have a lower risk of delayed motherhood compared to those from older cohorts [39] observed a general drop in the risk of delayed motherhood in newer birth cohorts. This trend is consistent across most demographic characteristics. However, some groups do not follow this pattern. Specifically, individuals with secondary or higher education, Scheduled Tribe (ST) groups, Christians and those of other religions, women who married at age 21 or older, women with no previous children [49] explain that childless women plan to have another child in future or more than three previous children, and those from the North East region do not exhibit a clear decline in the risk of delayed motherhood in newer cohorts similarly some of social norm and expectation [50, 51].

For urban, educated, and wealthier women, where delayed motherhood is often a result of career aspirations and delayed marriage rather than lack of awareness, interventions should focus on workplace support and reproductive counselling. Policies promoting flexible work hours, paid parental leave, and affordable childcare can help align reproductive intentions with biological feasibility [10, 29]. Furthermore, integrating fertility awareness programs into higher education and corporate wellness initiatives can support informed reproductive decision-making without stigmatizing delayed childbearing [27, 28].

In contrast, for rural, less-educated, and socioeconomically disadvantaged women particularly among Scheduled Castes and Tribes where access to reproductive health services and information remains limited, the priority should be on strengthening primary healthcare systems. Community-based reproductive health education, delivered through Accredited Social Health Activists (ASHAs) and rural health clinics, can improve awareness of fertility windows and risks associated with very late childbearing [48]. Programs like the National Health Mission (NHM) should incorporate modules on reproductive life planning, especially for women approaching their late 30 s.

Additionally, assisted reproductive technologies (ART) should be made more accessible and affordable, particularly for women over 35 facing infertility, through inclusion in public health insurance schemes like Ayushman Bharat [44]. However, such services must be accompanied by ethical counselling to counter the misconception that ART can fully reverse age-related fertility decline [5].

Finally, mass media and digital platforms can be leveraged to disseminate accurate, culturally sensitive information about fertility and maternal health. Given the high penetration of mobile phones and social media, even in rural areas, targeted campaigns via WhatsApp, YouTube, and regional television can bridge knowledge gaps across the urban–rural divide [52].

### Strengths and limitations

This study leverages nationally representative NFHS data across five rounds, enabling robust, long-term trend analysis of delayed motherhood in India. The use of joinpoint and Age-Period-Cohort (APC) models allows for a nuanced disentanglement of age, period, and cohort effects, enhancing the depth of temporal insights. A large sample of 413,887 women aged 40–49 ensures high statistical power and generalizability. The study uniquely focuses on delayed *last* birth, contributing to under-researched areas in Indian fertility transitions. However, the cross-sectional design limits causal inference, relying on retrospective reporting prone to recall bias. Defining delayed motherhood as childbirth at ≥ 35 years, while clinically relevant, may not capture the full reproductive trajectory. The APC model, despite using the Maximum Entropy method, still faces inherent identification challenges. Findings may not generalize to younger, still-reproducing cohorts. Subgroup estimates (e.g., Northeast, high parity) suffer from smaller sample sizes and instability. Rural–urban and socioeconomic differences in awareness are acknowledged but not directly measured. The analysis is confined to ever-married women, excluding single or childless individuals. Despite these limitations, the study offers critical insights into India’s evolving fertility landscape.

## Conclusion

This study reveals significant shifts in delayed motherhood patterns in India between 1981 and 2010, with a notable decline in prevalence since the 1990 s, particularly among rural and less-educated women. Joinpoint and Age-Period-Cohort analyses highlight the complex interplay of age, period, and cohort effects, with newer cohorts generally exhibiting lower risks of delayed motherhood — though this trend is less evident among women with higher education, those from Scheduled Tribes, Christians, and residents of the Northeast. The findings highlights that delayed motherhood is not a uniform phenomenon but one shaped by socioeconomic status, education, geography, and cultural context. Therefore, public health responses must be equally nuanced. For urban, educated women, policies should support reproductive autonomy through work-family balance initiatives and fertility counseling. For rural and marginalized populations, the focus should be on improving access to reproductive health education and services. Region-specific strategies, especially for the Northeast, are needed to address persistent disparities. Addressing the challenges of delayed motherhood in India requires equitable, evidence-based, and multi-sectoral interventions that recognize both the biological risks and the social determinants of reproductive timing. Strengthening reproductive health systems, expanding access to fertility care, and promoting informed decision-making across all socioeconomic strata are essential steps toward ensuring safer motherhood and improved child health outcomes in the decades ahead.

## Supplementary Information

Supplementary Material 1.

## Data Availability

NFHS data is a nationally representative data set which is freely available in public domain.
