# Supplementary material for: Exploring the shifting landscape of delayed motherhood in India: a comprehensive analysis using joinpoint and age-period-cohort analysis
Source: BMC Womens Health. 2025 Nov 19;25:563. doi: 10.1186/s12905-025-04104-4 (PMC12628530; doi:10.1186/s12905-025-04104-4)
Supplement: Supplementary file 1 — Supplementary Material 1. [file 12905_2025_4104_MOESM1_ESM.docx]

**Supplementary Material**

**Figure 1:** **Temporal** **trends in the Prevalence of Delayed Motherhood by background characteristics in India from 1981 to 2010 using the Joinpoint Regression Analysis**

| 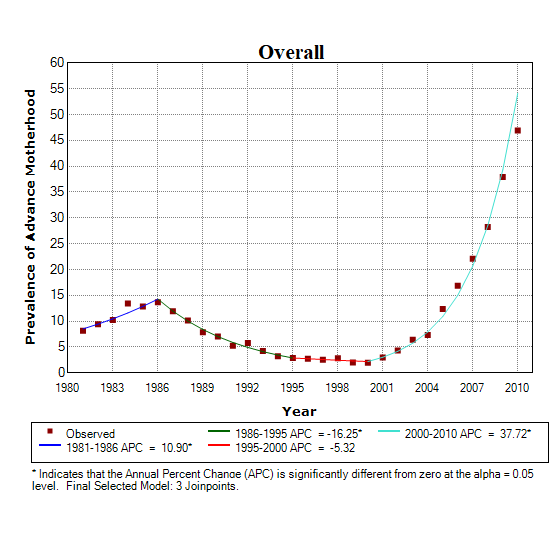 | 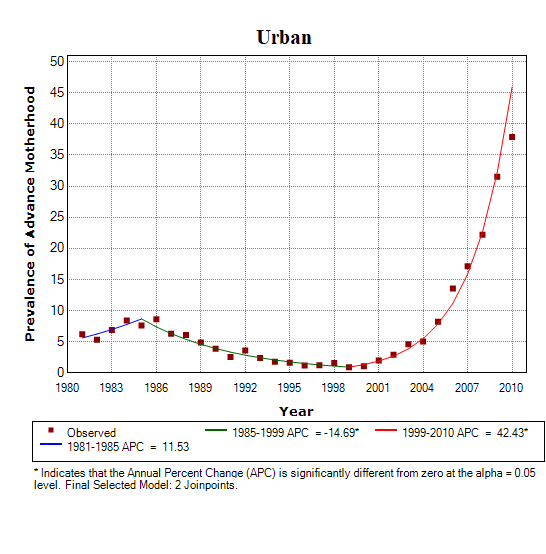 | 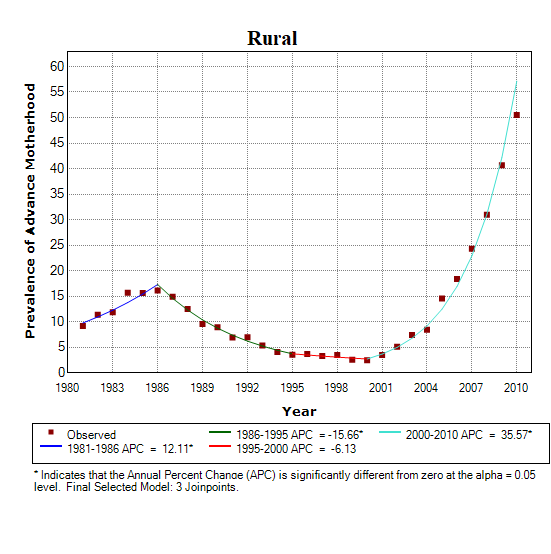 |
| --- | --- | --- |
| 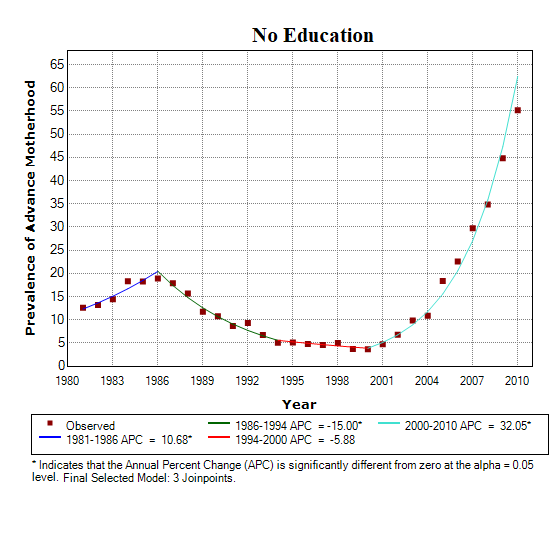 | 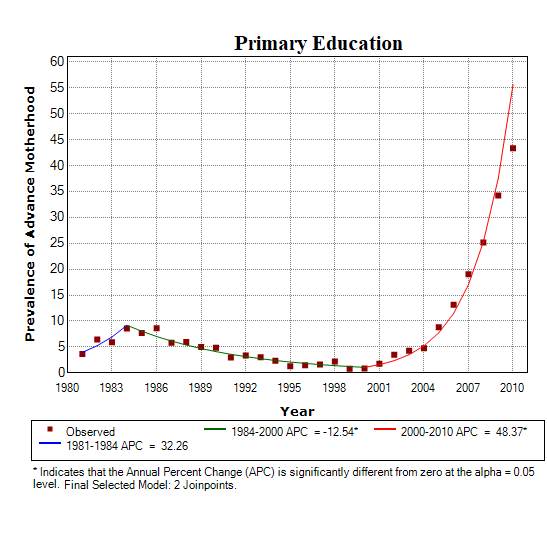 | 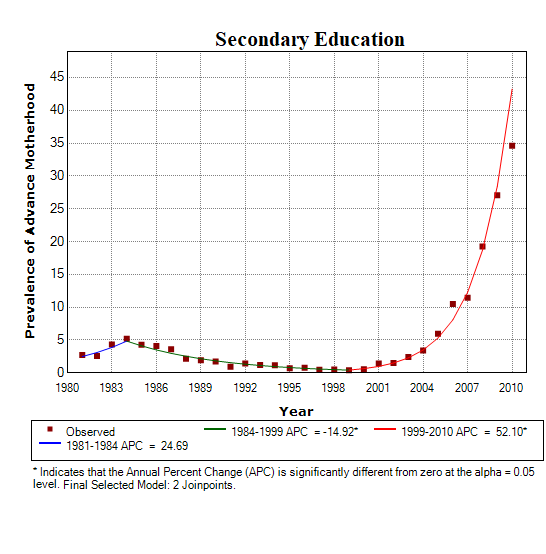 |
| 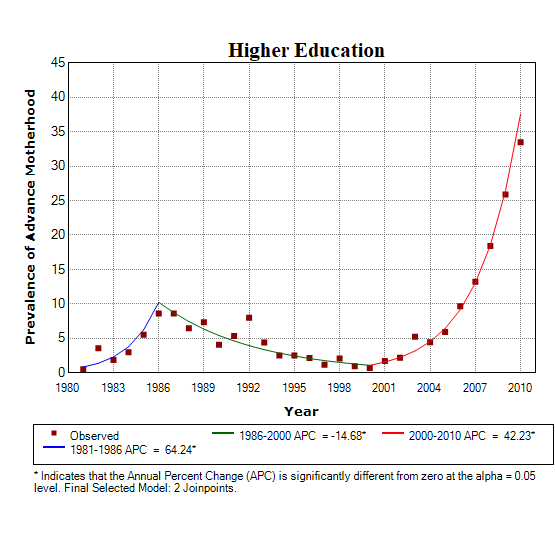 | 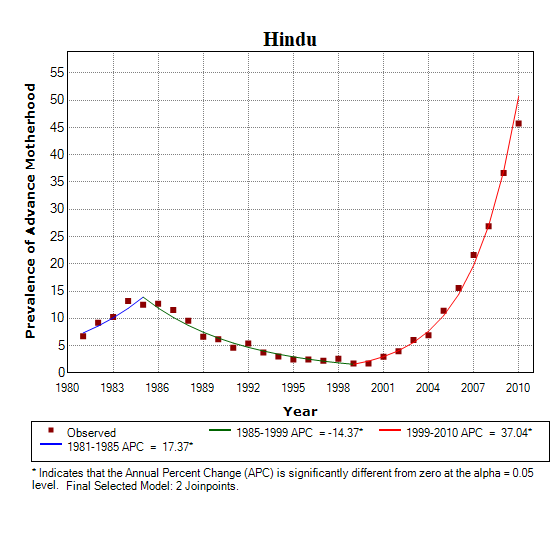 | 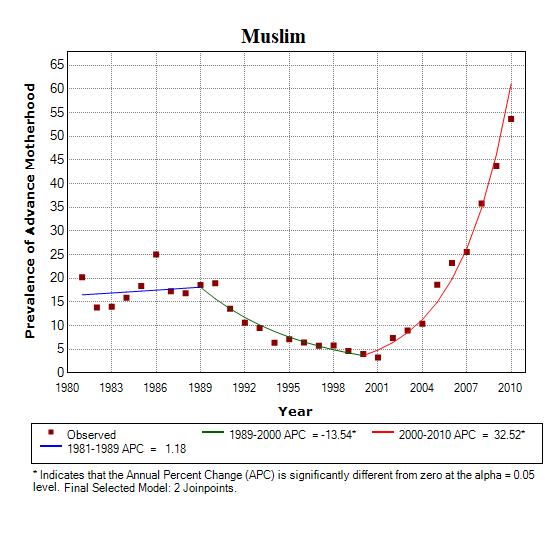 |
| 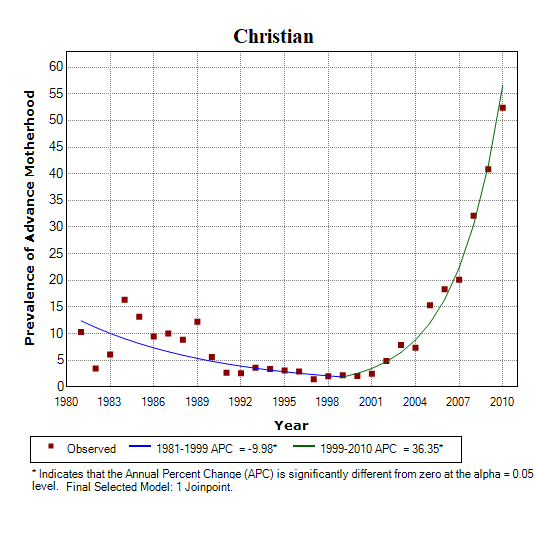 | 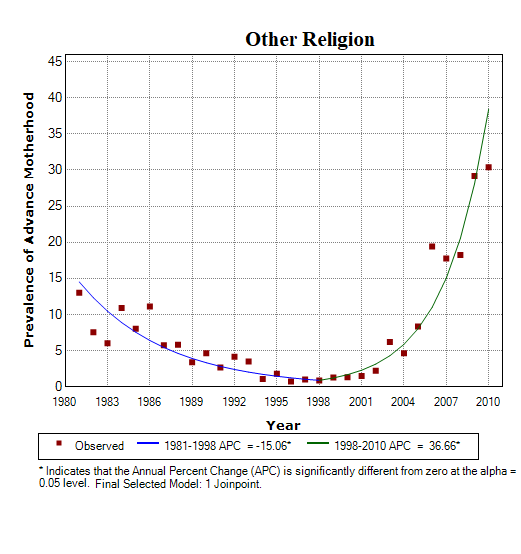 | 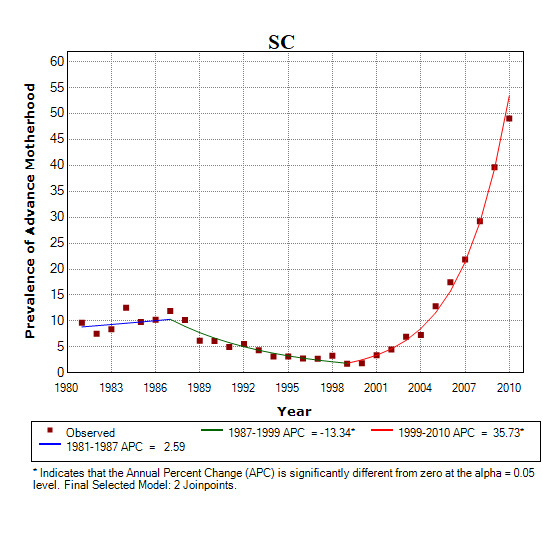 |
| 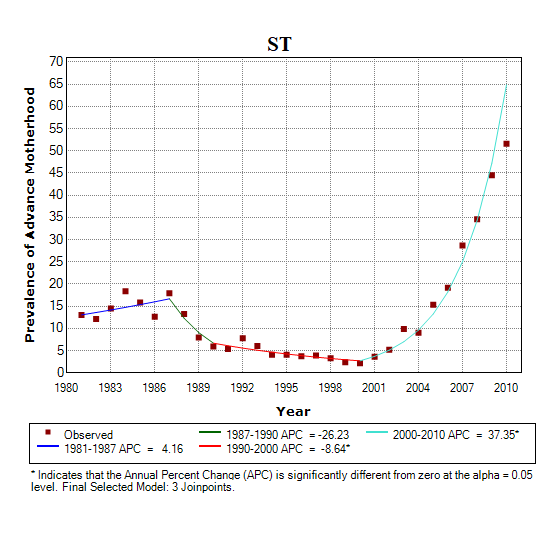 | 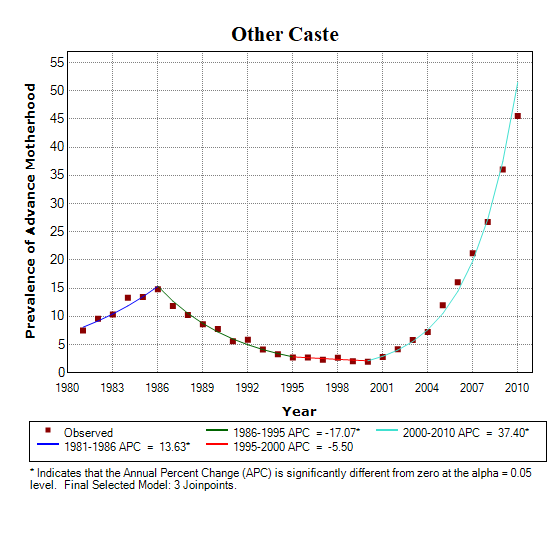 | 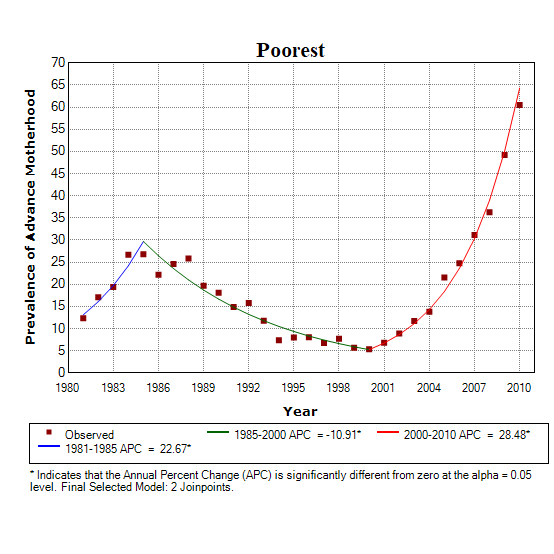 |
| 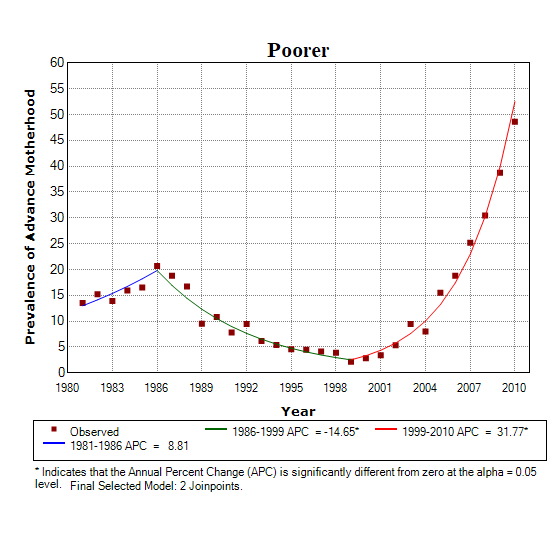 | 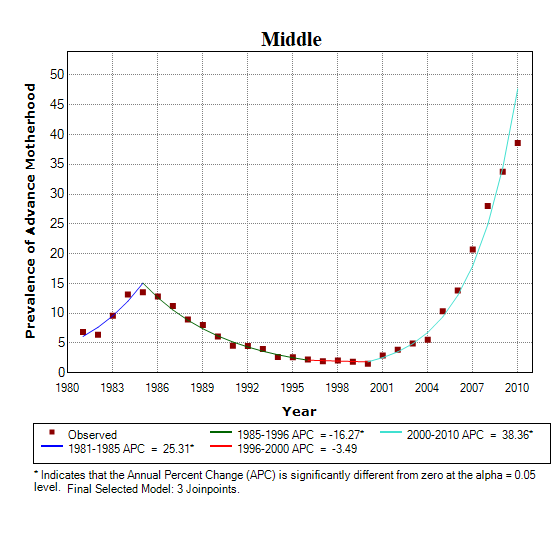 | 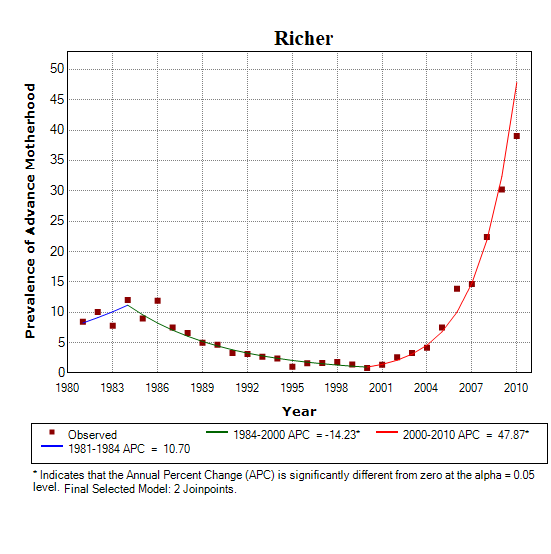 |
| 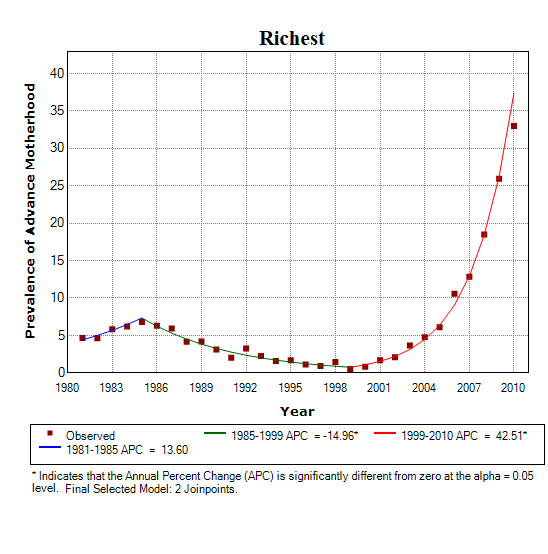 | 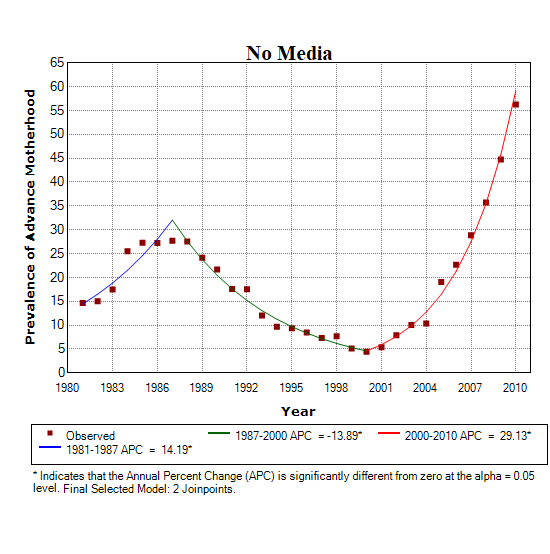 | 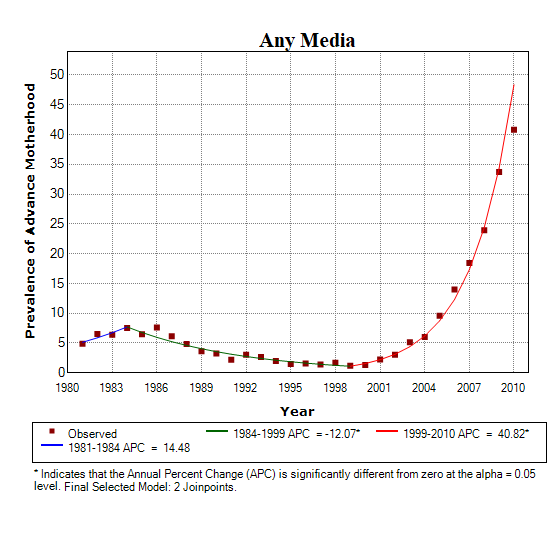 |
| 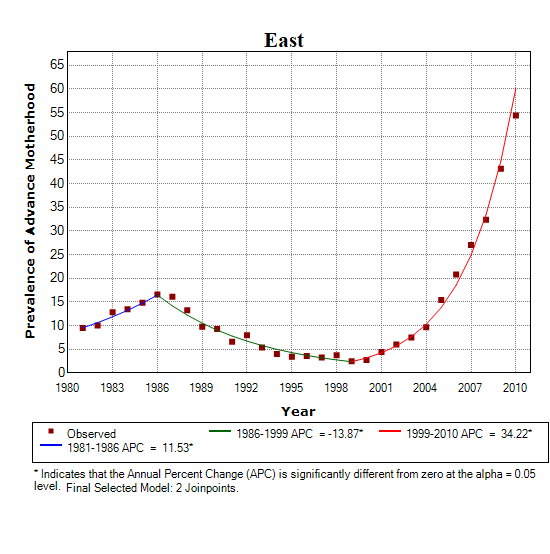 | 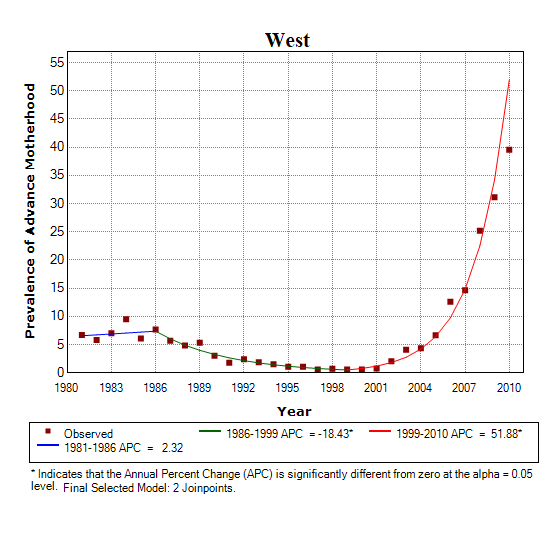 | 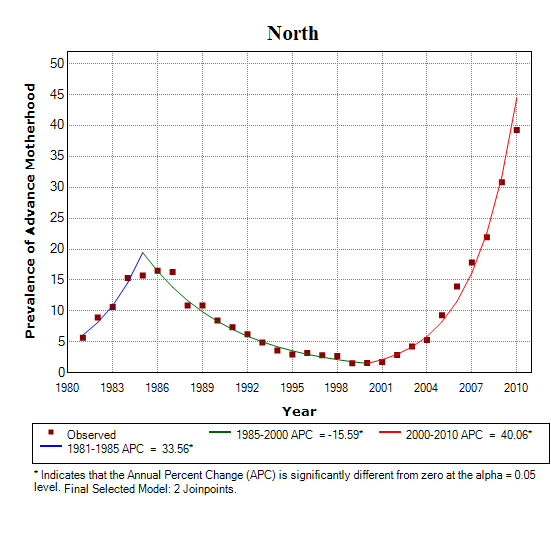 |
| 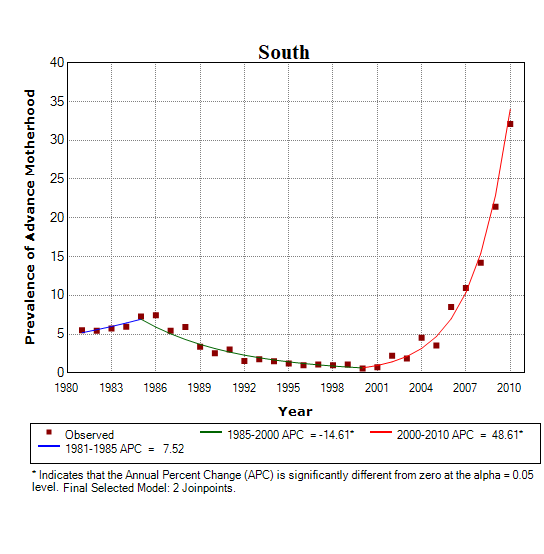 | 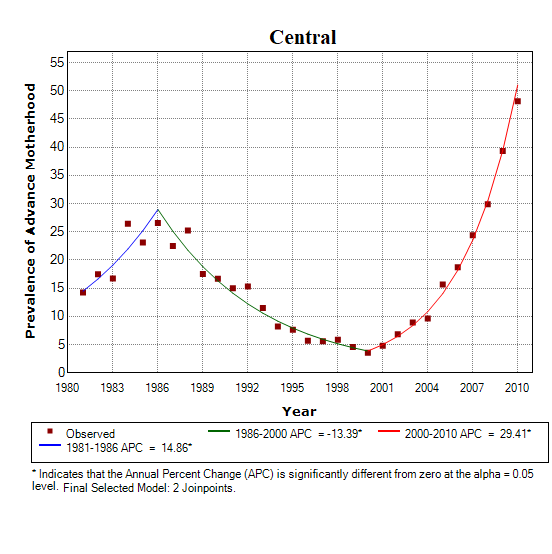 | 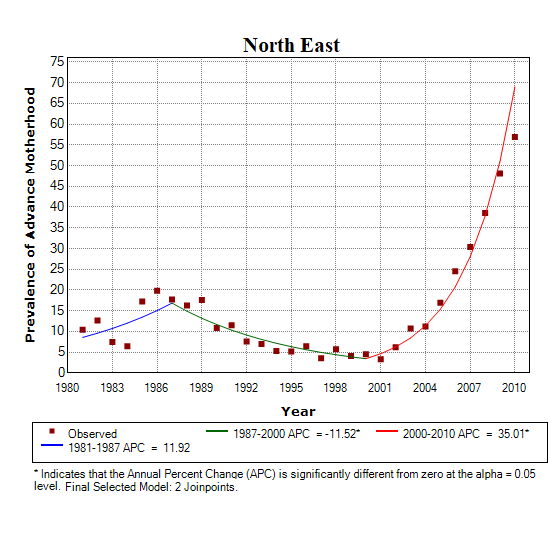 |
| 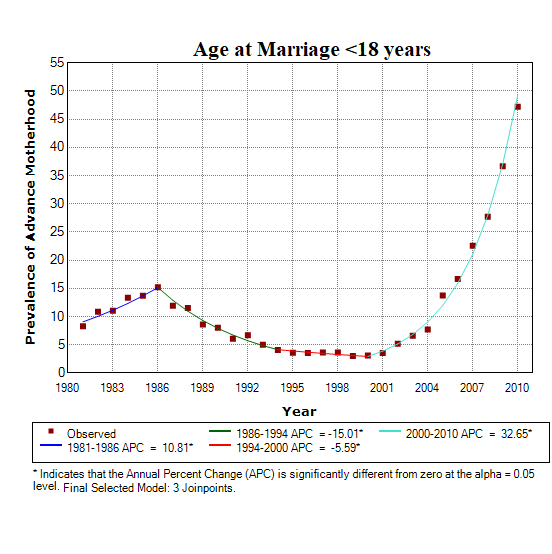 | 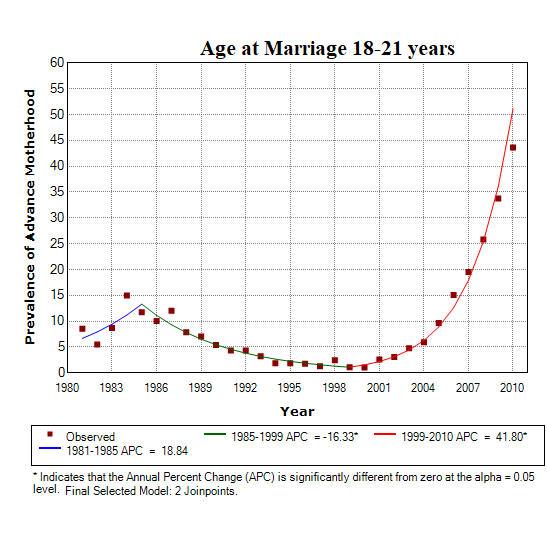 | 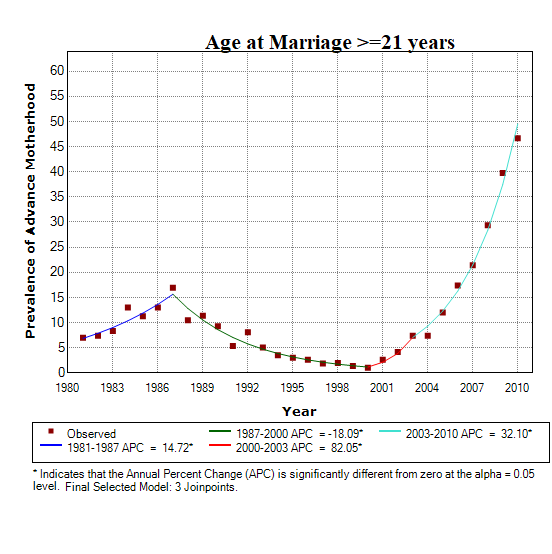 |
| 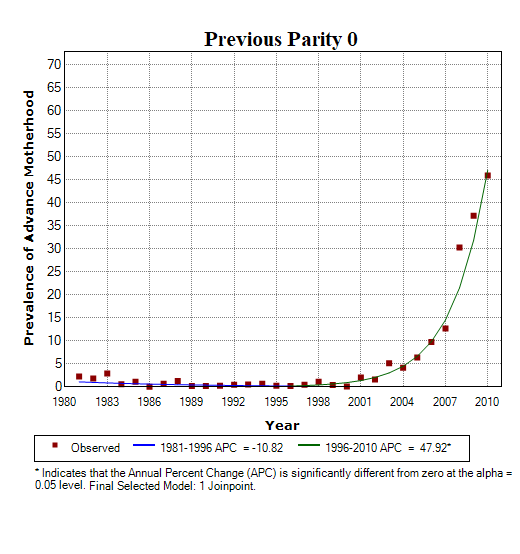 | 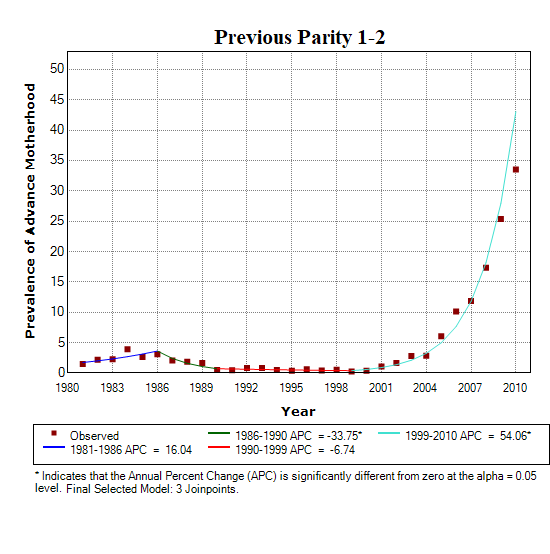 | 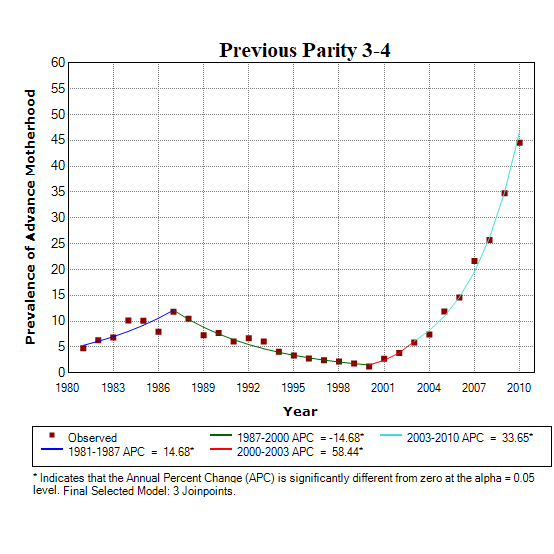 |
| 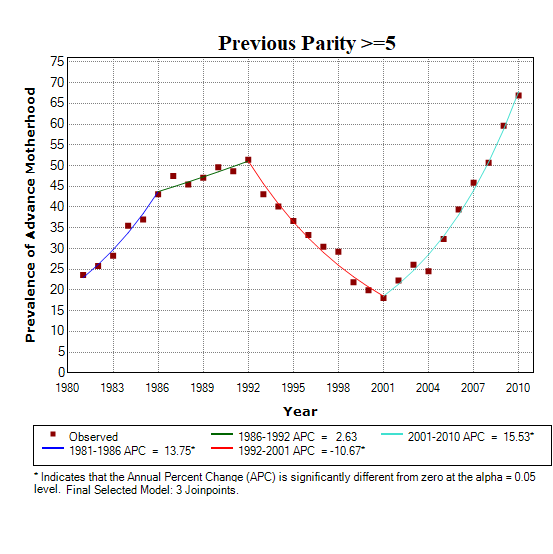 |  |  |
